# The Effects of Arsenic Trioxide on DNA Synthesis and Genotoxicity in Human Colon Cancer Cells

**DOI:** 10.3390/ijerph7052018

**Published:** 2010-04-28

**Authors:** Jacqueline J. Stevens, Barbara Graham, Alice M. Walker, Paul B. Tchounwou, Christian Rogers

**Affiliations:** 1 Molecular and Cellular Biology Research Laboratory, NIH-Center for Environmental Health, College of Science, Engineering and Technology, Jackson State University, 1400 JR Lynch Street, Box 18540, Jackson, MS 39217, USA; 2 Molecular Toxicology Research Laboratory, NIH-Center for Environmental Health, College of Science, Engineering and Technology, Jackson State University, 1400 JR Lynch Street, Box 18540, Jackson, MS 39217, USA; E-Mails: barbara.e.graham@jsums.edu (B.G.); alice.m.walker@jsums.edu (A.M.W.); paul.b.tchounwou@jsums.edu (P.B.T.); christian.s.rogers@jsums.edu (C.R.)

**Keywords:** Arsenic trioxide, HT-29 cells, [^3^H]thymidine incorporation assay, genotoxicity, comet assay

## Abstract

Colon cancer is the third leading cause of cancer-related deaths worldwide. Recent studies in our laboratory have demonstrated that arsenic trioxide is cytotoxic in human colon cancer (HT-29), lung (A549) and breast (MCF-7) carcinoma cells. The purpose of the present study is to investigate the effects of arsenic trioxide on DNA synthesis and the possible genotoxic effects on human colon cancer cells. HT-29 cells were cultured according to standard protocol, followed by exposure to various doses (0, 2, 4, 6, 8, 10, and 12 μg/mL) of arsenic trioxide for 24 h. The proliferative response (DNA synthesis) to arsenic trioxide was assessed by [^3^H]thymidine incorporation. The genotoxic effects of arsenic-induced DNA damage in a human colon cancer cell line was evaluated by the alkaline single cell gel electrophoresis. Results indicated that arsenic trioxide affected DNA synthesis in HT-29 cells in a biphasic manner; showing a slight but not significant increase in cell proliferation at lower levels of exposure (2, 4 and 6 μg/mL) followed by a significant inhibition of cell proliferation at higher doses (*i.e.*, 8 and 10 μg/mL). The study also confirmed that arsenic trioxide exposure caused genotoxicity as revealed by the significant increase in DNA damage, comet tail-lengths, and tail moment when compared to non-exposed cells. Results of the [^3^H]thymidine incorporation assay and comet assay revealed that exposure to arsenic trioxide affected DNA synthesis and exhibited genotoxic effects in human colon cancer cells.

## 1. Introduction

Colon cancer is defined as any malignant neoplasm arising from the inner lining of the colonic epithelium, and is the third most common cancer and the leading cause of cancer related deaths for both men and women in the United States [1]. The majority of colon cancers are benign lesions (adenomas) that can develop into malignant tumors that span an average of 15–20 years. Colon cancer begins in the epithelial tissue of the colon, and the cancer spreads to nearby lymph nodes and other parts of the body during advance stages of development [1]. Early detection and yearly check-ups are known to reduce the risk of dying of colon cancer. Individuals with colon cancer can receive treatment and possibly be cured if the polyps are detected before the polyps become cancerous or finding the cancer at an early stage.

Arsenic is a well known human toxicant and carcinogenic metalloid. Exposure to arsenic and its compounds can have adverse effects on human health [2]. Epidemiological studies based on ingestion of arsenic have been implicated in noncarcinogenic health effects in various organs and systems including cardiovascular, dermal, reproductive, neurological, respiratory, hepatic, hematological, renal, and gastrointestinal [2]. Nonetheless, exposure to arsenic has been associated with the development of malignancies, severe gastrointestinal toxicities, diabetes, cardiovascular diseases, and even death. The relationship of arsenic and malignancy is a major concern. It has been documented that long-term arsenic exposure is associated with increased risks of numerous human cancers [2,3], and as a result, arsenic has been classified as a human carcinogen by both the U.S. Environmental Protection

Agency [4] and the IARC [5]. Exposure to arsenic orally or by inhalation can lead to increased risk of skin and lung cancer respectively [4]. In addition to lung cancer, cancers of the liver, kidney, lung, colon and bladder are affected with increased arsenic exposure [4,6,7]. The mechanism of action of arsenic in carcinogenesis has not been clearly elucidated [3,4]. Many mechanisms have been implicated for arsenic-induced carcinogenesis, such as genotoxicity, induction of oxidative stress and DNA damage, inhibition of DNA repair enzymes involved in nucleotide excision repair and base excision repair, tumor promotion, cell proliferation, chromosomal aberrations, and signal transduction or altered DNA methylation [8–12]. There is no ideal animal model to study arsenic-induced cancers observed in humans, therefore investigations using human cell lines and affected individuals in exposed areas are ongoing.

Arsenic toxicity has become a global public health concern and has affected human health. Numerous epidemiological studies have reported that large populations in the world are being exposed chronically to arsenic [4]. Exposure to inorganic arsenic occurs via environmental (e.g., contaminated drinking water, air, food, domestic fuel sources) and occupational exposures (e.g., smelting industries, pesticide production). The toxicity of arsenic is very complicated and the toxicity varies on its oxidative state and solubility [4,13,14]. The trivalent compounds such as arsenic trioxide, sodium arsenite and arsenite, and arsenic trichloride are more toxic than the pentavalent compounds such as arsenic pentoxide, arsenic acid, lead and calcium arsenates [4,13]. The trivalent and pentavalent forms of arsenic are found in arsenic-contaminated water [13,15]. This metalloid affects a variety of tissues and cells depending on the degree of exposure. Acute or chronic exposure has been reported in several countries around the world due to the high concentrations found in drinking water [6,16–19]. The increased cancer risk posed by contaminated water is thought to be due to the presence of inorganic trivalent arsenite [20]. The impairment of cellular respiration by the inhibition of various mitochondrial enzymes, and the uncoupling of oxidative phosphorylation is believed to be one of the mechanisms by which arsenic exerts is toxic effect [4]. Arsenic toxicity is thought to result from its ability to interact with sulfhydryl groups of proteins and enzymes, and to substitute phosphoruous in a variety of biochemical reactions [4,14]. Research has shown that the major metabolic pathway for inorganic arsenic in humans is methylation in which most of the inorganic arsenic is metabolized to monomethylarsonic acid and dimethylarsinic acid before excretion in the urine [4]. There is still much discussion as to whether arsenic is a carcinogen by itself or acts synergistically to predispose cells to transformation by complete carcinogens [10].

Tests for genotoxicity have indicated that arsenic compounds inhibit DNA repair, induce chromosomal aberrations, and sister chromatid exchanges [4,21–23] Genotoxic effects of arsenic have been reported in various cell lines and rodents [24–26]. Studies have shown that arsenic acts through a series of chemical reactions in the cell, interacting strongly with nearby molecules, and changing the structure of cellular components such as DNA [24]. Arsenic does not induce genetic mutations in most test systems, but chromosomal damage has been reported in cultured mammalian cells, possibly as a result of arsenic’s effects on the enzymes involved in DNA replication and repair. Arsenic has been implicated in promoting alterations in growth and proliferation pathways, apoptotic pathways, DNA repair mechanisms, and stress-response pathways [27–29]. Some studies have reported that the generation of DNA damage includes DNA strand breaks [30]. Recent studies have provided evidence that arsenite can induce DNA damage by promoting the formation of reactive oxygen species particularly superoxide radical anions and hydrogen peroxide [8–9]. Arsenical compounds have also been shown to induce gene amplification, arrest cells in mitosis, and inhibit gene repair [4,31]. The mechanism of genotoxicity is not known, but it may be due to the ability of arsenate to inhibit DNA, replicating or repair enzymes [6,32].

Arsenic compounds have been used in a variety of beneficial applications. These compounds have been used in industry for the production of paints and glass and are components of semiconductors. In addition, arsenic compounds are also used to manufacture products with agricultural applications such as insecticides, fertilizers, weed kills, fungicides, and wood preservatives [6]. Arsenic has a long history of usage as a medicinal. Many arsenic-containing compounds have been used for treatment of human diseases such as syphilis, amoebic dysentery, African sleeping sickness, and Hodgkin’s lymphoma [4,33]. Although arsenic trioxide (As_2_O_3_) is highly toxic, this compound has been shown to have therapeutic potential. Inorganic arsenic trioxide has been clinically approved for the treatment of several hematological malignancies [33]. Studies have shown that arsenic trioxide has antitumor properties and has been approved by the Food and Drug Administration (FDA) for the treatment of promyelocytic leukemia in humans [34,35]. In addition, As_2_O_3_ has been shown to inhibit proliferation, induce apoptosis, and inhibit angiogenesis in multiple myeloma cells *in vitro* and *in vivo* [36]. Some studies have suggested that As_2_O_3_ may have activity in the treatment of some solid tumor such as lung cancer, breast cancer, esophageal carcinoma, ovarian, and gastric cancer. Studies have investigated the anti-vascular effect of As_2_O_3_ and the potential of combining As_2_O_3_ with radiation therapy [37]. Significant benefits of arsenic trioxide have been reported, which further improved the outcome of patients with these malignancies.

There is no ideal treatment for colon cancer. One of the primary issues for cancer chemotherapy is how to kill cancer cells selectively without damaging normal cells. Arsenic trioxide may be useful in combination therapy. It has been reported that the combination therapy of As_2_O_3_ with L-buthionine-sulfoximine enhanced *in vitro* growth inhibition effect of As_2_O_3_ on prostate, breast, lung, colon, cervix, bladder, and kidney cancers compared with As_2_O_3_ alone [38]. The aim of this present study was to use human colon cancer (HT-29) cells as an *in vitro* test model to evaluate the potential therapeutic action of arsenic trioxide against colon cancer. We hypothesized that As_2_O_3_ is cytotoxic to HT-29 cells and its cytotoxicity is associated with DNA damage in cancer cells.

## 2. Materials and Methods

### Reagents and Cell Line

Human colon cancer (HT-29) cells were purchased from American Type Culture Collection (ATCC) (Manassa, VA). Arsenic trioxide with 99.9% purity was purchased from Fisher Scientific (Houston, TX). McCoy’s 5A growth medium, and 0.4% trypan blue were purchased from ATCC (Manassas, VA). Fetal bovine serum (FBS), penicillin/streptomycin, phosphate buffered saline (PBS), and 0.25% trypsin-EDTA (w/v) were purchased from Gibco (Grand Island, NY). [^3^H methyl]Thymidine was obtained from MP Biomedicals (Irvine, CA). CometAssay^TM^ kit was purchased from Trevigen, Inc (Gaithersburg, MD).

### Cell Culture

HT-29 cells were maintained in complete growth medium (CGM) [McCoy’s 5A medium supplemented with 10% FBS and 1% antibiotics (penicillin/streptomycin)]. HT-29 cells (1 × 10^6^ cells/mL) were plated in T-25 flasks containing 5 mL of CGM and grown in a humidified incubator under an atmosphere of 95% air and 5% CO_2_ at 37 ºC to sub-confluence (90–95%). The culture medium was replaced every 48 h. Once the cells reached 90–95% confluency, the medium was aspirated, and the cell monolayer was washed three times with sterile phosphate buffered saline. The cell monolayer was treated with 1 mL of 0.25% (w/v) trypsin-EDTA and incubated briefly at 37 ºC and visualized microscopically to ensure complete cell detachment. Cells were re-suspended in McCoy’s 5A complete growth medium. For experimental purposes, cells were stained with trypan blue, incubated for 2 min at room temperature, and counted using a hemacytometer. Cells were seeded on 13 × 100 mm tissue culture plates at a density of 1 × 10^5^ cells/plate prior to arsenic trioxide treatment.

### [^3^H]Thymidine Incorporation Assay

Incorporation of [^3^H]thymidine into newly synthesized DNA was used as a measure of cell proliferation. Cells were seeded in 6-well tissue culture plates at a density of 5 × 10^5^ cells/well in CGM and grown at 37 ºC in a 5% CO_2_ humidified incubator until cells were 60%–70% confluent. Sub-confluent cells were incubated overnight in McCoy’s 5A medium supplemented with 1% fetal bovine serum and 1% antibiotics (penicillin/streptomycin) prior to treatment. Cells were treated with different doses of arsenic trioxide (0, 2, 4, 6, 8, 10, and 12 μg/mL) in CGM. The control (0 μg/mL) received complete growth medium only. Cells were incubated for 18 h before 1μCi/mL [^3^H methyl]thymidine was added to each well for an additional 6 h period to measure the incorporation of [^3^H methyl] thymidine into the DNA. The incubation of tritiated thymidine was terminated by aspirating the culture medium and followed by three washes of cold phosphate buffered saline pH 7.4. In addition, 2 mL of ice cold 10% trichloroacetic acid (TCA) was added to each well and incubated for 20 min at 4 °C. The TCA was aspirated and the cells were washed three times with ice-cold water. After the washing, the cells were solubilized with 1 mL of 0.5 M NaOH/well at 37 °C for 30 min. [^3^H]Thymidine incorporation was quantitated in a scintillation counter (Beckman Instruments). Each treatment group was assayed in triplicates, and each experiment was repeated at least three times.

### Genotoxicity Study

Comet assay kit was used according to the instructions of the manufacturer (Trevigen Inc, Gaithersburg, MD). HT-29 cells were seeded at 3 × 10^5^ per plate and placed at 37 ºC in a 5% CO_2_ humidified incubator until 70% confluency. The CGM was removed, and the sub-confluent cells were incubated overnight in McCoy’s 5A medium supplemented with 1% FBS and 1% antibiotics (penicillin/streptomycin) for 24 h prior to treatment. Cells incubated in CGM served as a control for DNA damage (untreated cells). Cells were treated with different doses of As_2_O_3_ (0, 2, 4, 6, 8, 10, and 12 μg/mL) for 24 h in complete growth medium. Following the As_2_O_3_ treatments, the medium was removed, and the cells were washed three times with cold PBS, trypsinized with 1 mL of 0.25% trypsin-EDTA, harvested, and counted. The cells were spun down at 3000 rpm for 5 min. The pellet was re-suspended in PBS at a cell density of 1 × 10^5^. The cells were combined with molten LMAgarose (at 37 ºC) at a ratio of 1:10 (v/v), and 75 μL was immediately pipetted onto CometSlide^TM^. The slides were placed flat in a refrigerator at 4 °C for 30 min, and then immersed in prechilled lysis solution on ice for 1 h. Excess buffer from slides was removed, and the slides were immersed in freshly prepared alkaline solution, pH > 13 (0.6 g of NaOH pellets, 250 μL of 200 mM EDTA and 49.75 mL of dH_2_O) for 1 h. Slides were washed twice for 5 min with 1X TBE electrophoresis buffer (Tris base, boric acid, and EDTA) and electrophoresed in a horizontal gel apparatus at 1 V/cm (22 V) for 10 min. Slides were placed in 70% ethanol for 5 min, removed, and tapped to remove excess ethanol. Slides were air dried overnight, stained with SYBR Green, and allowed to set for 12 h. Photographs were taken to illustrate the changes in DNA morphology associated with arsenic trioxide exposure. For examining SYBR Green stained comet slides, a total of 150 comets were scored per arsenic trioxide dose. Seventy-five comets were randomly selected from three replicated slides, viewed with an Olympus Epifluorescence Microscope, and analyzed using the LAI’s Automated Comet Assay Analysis System (LACAAS) (Loates Associates, Inc. Westminister, MD). The experiment was repeated at least three times.

### Statistical Analysis

#### [^3^H]Thymidine Incorporation Assay

Experimental data were represented as means ± SEMs since each experiment was repeated three times. Statistical analysis was performed uising SAS software (SAS Institute, Cary, NC) for Microsoft Windows. The Dunnett’s test was used to determine statistical differences in DNA synthesis between control and arsenic trioxide-treated HT-29 cells. Multiple comparisons were done by ANOVA. All *P*-values < 0.05 were considered to be significant.

#### Genotoxicity Study

Data generated from the Comet assay were analyzed using the LAI’s Automated Comet Assay Analysis System (LACAAS) that provided the mean values and standard deviations of percent DNA damage, tail length, and tail moment. Similar to the DNA incorporation data, ANOVA was applied for global comparison followed by Dunnett’s test for identifying groups whose means were significantly different from the control.

## 3. Results

### [^3^H]Thymidine Incorporation Assay

**Figure 1.**
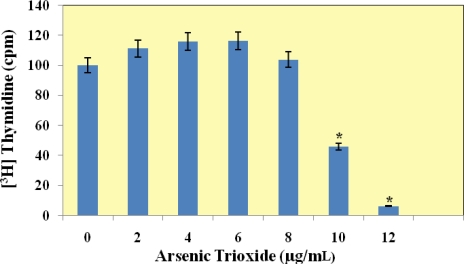
[^3^H]thymidine incorporation assay of HT-29 cells after 24 h exposure to arsenic trioxide. Cell proliferation was determined by measuring the [^3^H]thymidine incorporation (cpm) into DNA. Data are represented as mean ± SEM of three separate experiments performed in triplicates. Statistical analysis was done using the SAS software system. Differences were considered statistically significant with a *P* value < 0.05 according to the Dunnett’s test. The significance of the value is indicated by asterisks (*).

Data obtained from the [^3^H]thymidine incorporation assay exhibited a biphasic response to arsenic trioxide in the colon cancer cells. The results showed a slight but not significant increase in cell proliferation at lower levels of exposure (2, 4 and 6 μg/mL) followed by a significant inhibition of cell proliferation at higher doses (*i.e.*, 8 and 10 μg/mL) relative to the controls (Figure 1). The change in [^3^H]thymidine uptake and DNA incorporation was statistically significant at 8 and 10 μg/mL.

### Genotoxicity Study

**Figure 2.**
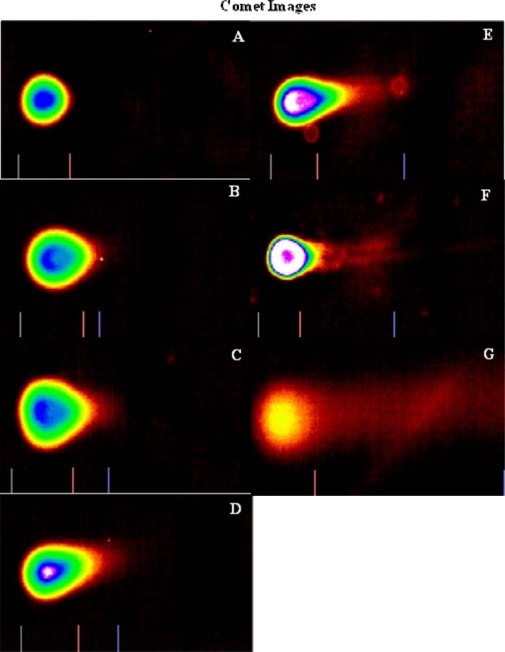
Representative comet assay images depicting the genotoxic effect of arsenic trioxide-treated (A = 0 μg/mL, B = 2 μg/mL, C = 4 μg/mL, D = 6 μg/mL, E = 8 μg/mL, F = 10 μg/mL, and G = 12 μg/mL) colon cancer cells exposed for 24 h using SYBR^®^ green staining.

The comet assay permits quantitative assessment of the effects of DNA damage or apoptosis. The applications of the comet assay include analysis of genotoxic activity, human, and environmental biomonitoring to DNA repair processes, cellular response to DNA damage, chromosomal damage, cancer risk assessment, and cancer cell resistance to treatment [39]. The genotoxic effect of arsenic trioxide was investigated in colon cancer (HT-29) cells. Figure 2 is the representative picture of comet images displaying the cell DNA migration patterns by the alkaline comet assay for HT-29 cells treated with arsenic trioxide for 24 h. As shown in this figure, the nuclear DNA of untreated cells is perfectly round and retains a highly organized association with matrix proteins in the nucleus. The nuclear DNA of arsenic-treated cells is severely fragmented as the dosage increases and the organization is disrupted. The individual strands of DNA lose their compact structure and relax and DNA fragments (damage) are observed. The extent of DNA damage was determined by measuring the tail moment, the percent of DNA fragmentation (percent of DNA in the comet tail *versus* total DNA), and the length of the comet tail. The higher the percent of DNA fragments and tail moment, the more severe is the damage. Similarly, the brighter and longer the comet tail, the higher the level of damage.

**Figure 3.**
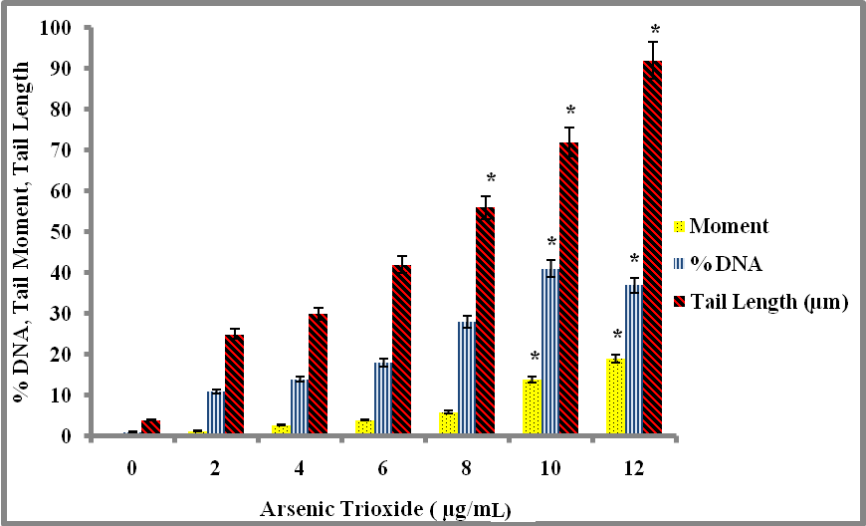
Arsenic trioxide-induced DNA damage as characterized by the increase in tail moment, the percentage of DNA damage, and the length of comet tails in colon cancer (HT-29) cells. Cells were exposed to arsenic trioxide for 24 h, and the comet assay was performed as described in the Materials and Methods section. Data were expressed as means ± SDs. Differences were considered statistically significant at *P* < 0.05 according to the Dunnett’s test.

The comet assay revealed that treatment of colon cells with arsenic trioxide caused severe damage to the cells. Results of the visual scoring of tail moment, the length of the comet tails, and total DNA damage are shown in Figure 3. The HT-29 cells were treated with 0–12 μg/mL of arsenic trioxide for 24 h, which resulted in an increase in tail moment in the treated cells compared with the control. DNA damage was significant as indicated by the increased tail moment, tail length, and the percentage of DNA fragmentation observed in the treated cells compared with the control. As a result, the DNA damage induced by the arsenic trioxide was dose-dependent.

## 4. Discussion

Colorectal cancer is one of the most commonly diagnosed cancers in the United States and is responsible for about 53,000 deaths. Great strides have been made in the treatment of colon cancer. It has been well documented that the environmental contaminant, inorganic arsenic, is a human toxicant and carcinogen. Epidemiological studies have demonstrated that chronic arsenic exposure causes tumors of the skin, urinary bladder, lung, liver, prostate, kidney, and colon [10,11]. Studies in arsenic carcinogenesis were first reported in cases of skin cancer after exposure to inorganic arsenic in drinking water [11,12]. Hwang *et al.* have shown that low levels of arsenic exposure predispose cells for malignant transformation [40]. Several potential mechanisms have been suggested such as induction of oxidative stress and DNA damage, stimulation of cell proliferation, inhibition of DNA repair, and deregulation of DNA methylation in arsenic-induced carcinogenesis [28,29]. However, the mechanism(s) underlying its carcinogenicity remains unclear. Arsenic has cytotoxic effects on several cancer cell lines. Reported studies in our laboratory have shown that As_2_O_3_ induces cytotoxicity in a dose-dependent manner in HT-29 cells [41]. Tchounwou *et al.* revealed a dose-response in human liver carcinoma cells (HepG_2_) [42] and human leukemia cells (HL-60) when exposed to arsenic toxicity [43]. It has been reported that As_2_O_3_ induced clinical remission in patients with acute promyelocytic leukemia and other malignancies.

Historically, cell proliferation assays relied on the detection of tritiated thymidine [^3^H] uptake. The thymidine incorporation assay measures the inhibition of deoxyribonucleic acid (DNA) synthesis by the proliferating cell population following exposure to a drug or chemical. In the present study, we demonstrated that arsenic trioxide affected DNA synthesis in colon cancer cells (HT-29). Data obtained from the [^3^H]thymidine incorporation assay exhibited a biphasic response to arsenic trioxide in the colon cancer cells. The data from this study reveals that arsenic trioxide inhibits cellular proliferation at higher doses (*i.e.*, 8 and 10 μg/mL). This result is consistent with preliminary studies in our laboratory that revealed a biphasic response to arsenic trioxide in the breast carcinoma (MCF-7) cell line. Studies have shown that arsenic trioxide is acutely toxic to both breast carcinoma (MCF-7) and lung carcinoma (A549) cell lines [44]. Arsenic trioxide has been shown to significantly enhance growth inhibition in human breast cancer MCF-7 cells determined by the MTT [3-(4,5-dimethylthiazol-2-yl)-2,5-diphenyltetrazolium bromide] assay and [^3^H]thymidine incorporation assay [45]. Hamadeh *et al.* demonstrated that the incorporation of [^3^H]thymidine was markedly increased, relative to the control levels in arsenite-exposed keratinocytes indicating an increase in cell proliferation [46]. Germolac *et al.* reported that human keratinocytes, treated with sodium arsenite resulted in a significant increase in cell proliferation as indicated by the incorporation of [^3^H]thymidine into cellular DNA [47]. Trouba *et al.* reported an increase in cell proliferation observed in human fibroblasts exposed to sodium arsenite [48]. Lee *et al.* showed that arsenic trioxide alone slightly inhibited the growth of HCT116 colon cancer cells, whereas the combination of arsenic trioxide and sulindac reduced cell growth by 30–40% [49]. Data in this study reveal that arsenic trioxide increases cellular proliferation in human colon cancer cells as measured by [^3^H]thymidine incorporation. These observations confirm the effects of arsenic on cell proliferation in keratinocytes, fibroblasts, human breast (MCF-7), and HCT116 colon cancer cells.

The comet assay, also known as single gel electrophoresis, is a visual and sensitive method used to measure single-strand (ss) DNA breaks or DNA cleavage in mammalian cells [30]. Any DNA damage is represented as a tail length (tail migration) of the DNA strand. The overall structure resembles a comet with a circular head corresponding to the undamaged DNA that remains in the cavity and a tail of damaged DNA. The damage usually detected by the comet assay is single-strand breaks and double strand breaks [50]. Arsenic has been shown to induce DNA damage in human cells. In our present study, we have observed molecular changes in HT-29 cells. DNA damage induced by arsenic trioxide in colon cancer cells was determined by alkaline comet assay. The cells exhibited a high degree of DNA damage after 24 h treatment as shown by the increase in tail moment, tail length, and percentage of DNA damage observed in the treated cells compared to the controls. There was a dose-dependent increase in the tail moment, tail length, and the percentage of DNA in the tail. The increase in DNA fragmentation is thought to lead to increase in apoptosis in cells treated with arsenic trioxide. Our studies are consistent with previous findings in that the increase in arsenic results in an increase in the level of DNA damage.

Studies have shown that arsenic trioxide is highly cytotoxic and genotoxic to human leukemia (HL-60) cells. These studies using the comet assay showed that arsenic trioxide induced DNA damage in a dose-dependent manner [43]. Graham *et al.* showed that arsenic was highly genotoxic to human keratinocytes, melanocytes, and dendritic cells [25]. The data showed a statistically significant dose-related increase in percentage of tail DNA. Studies have shown that arsenic-induced DNA damage in human hepatocytes measured by the comet assay [51]. Genotoxic studies have reported that arsenic trioxide induces DNA damage in lymphocytes [4]. Guillamet *et al.* have reported DNA damage in the human lymphobastoid cell line (TK6) by the alkaline Comet assay [52]. Several studies have implicated oxidative stress and free radical formation as important factors in the genotoxicity and cytotoxicity of arsenic compounds [8,9]. Arsenic trioxide exerts its effects mainly through elevated oxidative stress, but the exact molecular mechanism remains elusive. Reactive oxygen species (ROS) such as hydrogen peroxide, superoxide anion, singlet oxygen, and hydroxyl radical can directly or indirectly damage cellular DNA and protein [9].

## 5. Conclusions

Human exposure to arsenic, a toxic environmental pollutant, is associated with an increased incidence of human cancers. Epidemiologic studies have long supported a link between human exposure to inorganic arsenic and an increased incidence of cancers, which have led to intensive investigations of the underlying mechanism. However, the precise molecular and cellular mechanism by which arsenic causes cancer has not yet been established. These studies have implicated a number of mechanisms such as induction of oxidative stress and DNA damage, stimulation of cell proliferation, inhibition of DNA repair, and deregulation of DNA methylation in arsenic-induced carcinogenesis [14,15]. Our data suggest that As_2_O_3_ can significantly affect proliferation in HT-29 cells. This research is also the first to report investigating the *in vitro* effects of DNA damage in human colon cancer (HT-29) cells. The approach of using the comet assay is very sensitive to detect genetic damage in the cells. It is expected that these data in this study will serve as a foundation for furthering our knowledge of the effects of arsenic trioxide on human colon cancer cells and will contribute to a better understanding of arsenic-based chemotherapy.
